# Chronic hypoxia induces the activation of the Wnt/β-catenin signaling pathway and stimulates hippocampal neurogenesis in wild-type and APPswe-PS1ΔE9 transgenic mice *in vivo*

**DOI:** 10.3389/fncel.2014.00017

**Published:** 2014-02-10

**Authors:** Lorena Varela-Nallar, Macarena Rojas-Abalos, Ana C. Abbott, Esteban A. Moya, Rodrigo Iturriaga, Nibaldo C. Inestrosa

**Affiliations:** ^1^Centro de Envejecimiento y Regeneración (CARE), Departamento de Biología Celular y Molecular, Pontificia Universidad Católica de ChileSantiago, Chile; ^2^Centro de Investigaciones Biomédicas, Facultad de Ciencias Biológicas y Facultad de Medicina, Universidad Andrés BelloSantiago, Chile; ^3^Laboratorio de Neurobiología, Departamento de Fisiología, Facultad de Ciencias Biológicas, Pontificia Universidad Católica de ChileSantiago, Chile

**Keywords:** hypoxia, HIF-1α, hippocampus, Wnt signaling pathway, β-catenin, neurogenesis, Alzheimer’s disease

## Abstract

Hypoxia modulates proliferation and differentiation of cultured embryonic and adult stem cells, an effect that includes β-catenin, a key component of the canonical Wnt signaling pathway. Here we studied the effect of mild hypoxia on the activity of the Wnt/β-catenin signaling pathway in the hippocampus of adult mice *in vivo*. The hypoxia-inducible transcription factor-1α (HIF-1α) was analyzed as a molecular control of the physiological hypoxic response. Exposure to chronic hypoxia (10% oxygen for 6–72 h) stimulated the activation of the Wnt/β-catenin signaling pathway. Because the Wnt/β-catenin pathway is a positive modulator of adult neurogenesis, we evaluated whether chronic hypoxia was able to stimulate neurogenesis in the subgranular zone (SGZ) of the hippocampal dentate gyrus. Results indicate that hypoxia increased cell proliferation and neurogenesis in adult wild-type mice as determined by Ki67 staining, Bromodeoxyuridine (BrdU) incorporation and double labeling with doublecortin (DCX). Chronic hypoxia also induced neurogenesis in a double transgenic APPswe-PS1ΔE9 mouse model of Alzheimer’s disease (AD), which shows decreased levels of neurogenesis in the SGZ. Our results show for the first time that exposure to hypoxia *in vivo* can induce the activation of the Wnt/β-catenin signaling cascade in the hippocampus, suggesting that mild hypoxia may have a therapeutic value in neurodegenerative disorders associated with altered Wnt signaling in the brain and also in pathological conditions in which hippocampal neurogenesis is impaired.

## Introduction

Neurogenesis in the adult brain is mainly restricted to the subventricular zone (SVZ) of the lateral ventricles and the subgranular zone (SGZ) in the hippocampal dentate gyrus (Alvarez-Buylla and Garcia-Verdugo, 2002; Zhao et al., 2008a). In the SGZ, neural stem cells (NSCs) give rise to neuroblasts that mature into functional dentate granule neurons that are integrated into the preexisting hippocampal circuitry (van Praag et al., 2002; Zhao et al., 2006; Mathews et al., 2010). Increasing evidence indicates that neurogenesis is relevant for hippocampal functions, such as spatial learning, object recognition and memory (Reviewed in Deng et al., 2010; Koehl and Abrous, 2011; Marin-Burgin and Schinder, 2012).

Neurogenesis is modulated by different physiological stimuli such as running, exposure to environmental enrichment, learning and stress (Kempermann et al., 1997; Gould et al., 1998; van Praag et al., 1999; Dobrossy et al., 2003; Drapeau et al., 2007; Piatti et al., 2011; Song et al., 2012). Several signaling molecules have been determined to be essential for the maintenance, self-renewal and proliferation of NSCs and for the differentiation into fully functional neurons (Suh et al., 2009; Schwarz et al., 2012; Faigle and Song, 2013; Varela-Nallar and Inestrosa, 2013). The possibility to activate endogenous NSCs and stimulate the generation of new neurons in the adult brain could have therapeutic potential in pathological conditions in which neurogenesis is altered, such as mood disorders, schizophrenia and neurodegenerative diseases (Kaneko and Sawamoto, 2009; Winner et al., 2011; Petrik et al., 2012). It has been shown that exposure to low oxygen concentrations (or hypoxia) can stimulate the proliferation and differentiation of cultured embryonic and adult NSCs (Vieira et al., 2011). This effect has been associated with β-catenin (Mazumdar et al., 2010; Cui et al., 2011), a key component of the Wnt/β-catenin signaling pathway.

The Wnt/β-catenin cascade is initiated by the binding of a Wnt ligand to its receptor, Frizzled, and co-receptors, such as the low-density lipoprotein receptor-related protein 5 (LRP5) and LRP6 (Cadigan and Liu, 2006; Gordon and Nusse, 2006), which triggers the phosphorylation of the protein Dishevelled (Dvl), and inhibits the degradation of β-catenin, which in the absence of Wnt stimulation is phosphorylated in a multiprotein complex, ubiquitinated and degraded by the proteasome (Aberle et al., 1997; Liu et al., 2002). The stabilization of β-catenin results in its translocation into the nucleus where it binds to members of the T-cell factor (TCF) and lymphoid enhancer factor (Lef) family and activates the transcription of Wnt target genes (Logan and Nusse, 2004). The Wnt/β-catenin signaling pathway regulates several aspects of central nervous system development and also plays fundamental roles in the adult nervous system (Salinas and Zou, 2008; Inestrosa and Arenas, 2010) where it regulates synaptic assembly and plasticity (Ahmad-Annuar et al., 2006; Cerpa et al., 2008) and adult neurogenesis (Lie et al., 2005; Kuwabara et al., 2009; Karalay et al., 2011; Varela-Nallar and Inestrosa, 2013).

It was previously found that hypoxia increases β-catenin signaling in cultured neonatal hippocampal NSCs (Cui et al., 2011) and embryonic stem cells (ESCs) (Mazumdar et al., 2010). Under hypoxic conditions, the hypoxia-inducible transcription factor-1α (HIF-1α) directly binds to the promoters of the Lef1 and TCF1 genes (Mazumdar et al., 2010), therefore regulating the transcriptional activity of β-catenin. Moreover, it was determined that Wnt/β-catenin signaling is active in low oxygen regions in the adult brain, including the SGZ, suggesting an association between low oxygen and β-catenin signaling *in vivo* (Mazumdar et al., 2010). However, it has not been determined whether hypoxia modulates the activation of the Wnt/β-catenin signaling cascade in the hippocampus. Here, we assessed whether chronic exposure to hypoxia stimulates the activation of the Wnt/β-catenin signaling pathway, specifically in the hippocampus of adult mice, and also we studied whether this hypoxic condition could stimulate SGZ neurogenesis in adult wild-type mice as well as in a double transgenic mouse model of Alzheimer’s disease (AD).

## Materials and methods

### Animals and treatments

APPswe/PSEN1ΔE9 mice, which express the Swedish mutation of APP (K595N/M596L) and PS1 with the deletion of exon 9 (APP-PS1 mice stock #004462), were obtained from The Jackson Laboratory (Bar Harbor, Maine). All procedures involving experimentation on animal subjects were approved by the Bioethical Committee of the P. Catholic University of Chile. All animals had access to water and food *ad libitum*, in a 12:12 h light/dark cycle.

### Bromodeoxyuridine (BrdU) administration

A single dose of Bromodeoxyuridine (BrdU) (Sigma-Aldrich, St Louis, MO, USA) was injected i.p. at 100 mg kg^−1^.

### Hypoxic exposure

Animals were exposed to hypoxia (10% O_2_ at normal barometric pressure) by placement of a mice cage in a plexiglass chamber for 6–72 h. The hypoxic environment in the chamber was achieved by inflow of N_2_ gas. The hypoxic level was controlled by an oxygen controller (Pro-Ox model 110, BioSpherix, USA). Mice had free access to water and food *ad libitum* during the hypoxic exposure. Control animals were kept at normoxic condition (21% O_2_).

### Perfusion and postfixation

Animals were anesthetized (100 g ketamine + 10 g xylazine in 10 µl saline/g), and then transcardially perfused with saline, followed by 4% paraformaldehyde (PFA) in 0.1 M PBS. The brain was removed and placed in a vial with 4% PFA in PBS for 24 h at room temperature, dehydrated in 30% sucrose, and kept at 4°C until analysis.

### Tissue sectioning

Each mouse brain was sectioned on a cryostat in 12 sets of serial coronal sections of 40 µm thickness (Leica Microsystems, Wetzlar, Germany) and collected in ice-cold-PBS in multiwall dishes (Encinas and Enikolopov, 2008). Each set contained a representative sample of the whole hippocampus (Abbott et al., 2013).

### Immunofluorescence

Immunodetection of BrdU and neuronal markers in tissue sections was carried out as previously described (Abbott et al., 2013). Primary antibodies used were: rat anti-BrdU (Abcam), rabbit anti-Doublecortin (Cell Signaling Technology Inc., Beverly, MA, USA), monoclonal anti-NeuN (Millipore, Billerica, MA, USA) and rabbit anti-Ki67 (Abcam). As secondary antibodies, Alexa (Molecular Probes) and DyLight (Abcam) conjugated antibodies were used. BrdU and Ki67 positive cells were counted using a fluorescence microscope (Olympus BX51, Tokyo, Japan) as described (Abbott et al., 2013). Double-labeled sections were analyzed by confocal laser microscopy (Olympus FV 1000). Image analysis and *z*-projections were made with ImageJ software (NIH, USA).

### Immunoblotting

The hippocampus and cortex of treated and control mice were dissected on ice and either immediately processed or frozen at −150°C. Immunoblotting was performed as previously described (Varela-Nallar et al., 2009). Primary antibodies used were: mouse anti-Dvl3, mouse anti-β-catenin, mouse anti-c-myc, mouse anti-cyclin D1 and rabbit anti-β-tubulin (all from Santa Cruz Biotechnology, Santa Cruz, CA, USA) and mouse anti-rabbit anti-HIF-1α (Novus Biologicals, Littleton, CO, USA).

### Statistical analysis

Statistical analysis was performed using Prism 5 software (GraphPad Software Inc., San Diego, CA, USA). Statistical significance of differences was assessed using the non-paired Student’s *t*-test or ANOVA, and non-normally distributed data was analyzed using the Mann-Whitney test or Kruskal Wallis. *P* < 0.05 was considered significant.

## Results

### Chronic hypoxia induces the activation of the Wnt/β-catenin signaling pathway in the hippocampus of adult mice

**Figure 1:**
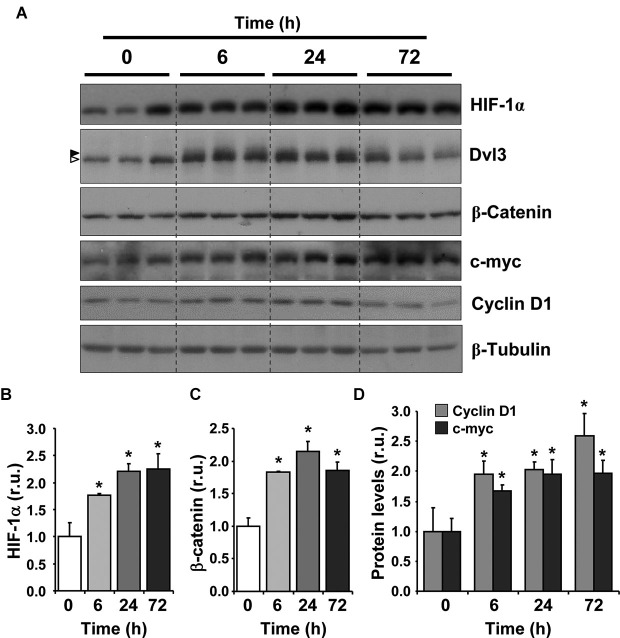
**Hypoxia induces the activation of Wnt/β-catenin signaling in adult mice. (A)** Immunoblot of total protein extracts from the hippocampus of 2-month-old mice exposed to hypoxia (10% O_2_) for 6, 24 and 72 h. Time 0 corresponds to control animals that were maintained at normoxic conditions (21% O_2_). Immunoblots of three different animals are shown in the control condition and in each time of exposure to hypoxia. In Dvl3 immunoblot, arrowheads on the left indicate dephosphorylated (white) and phosphorylated and shifted (black) Dvl3. **(B–D)** Densitometric analysis expressed in relative units (r.u.) of HIF-1α **(B)**, β-catenin **(C)**, cyclin D1 and c-myc **(D)** levels normalized to β-tubulin levels and compared to control mice that were not exposed to hypoxia. Bars represent mean ± S.E (*n* = 3 mice). * *p* < 0.05.

An association between low-oxygen and the transcriptional activity of β-catenin has been previously reported (Mazumdar et al., 2010); however, it is not known whether it also involves the activation of the Wnt/β-catenin signaling cascade. We aimed to determine whether hypoxia exposure could stimulate the activation of the Wnt signaling pathway in the hippocampus of adult mice *in vivo*. For this purpose, 2-month-old mice were placed in hypoxic chambers with 10% oxygen for 0 (normoxic control), 6, 24 or 72 h. After treatment animals were immediately sacrificed, the brain was removed and the hippocampus dissected and analyzed by immunoblot (Figure 1A). Hypoxia induced a significant increase in HIF-1α for all exposure times compared to the normoxic control (Figure 1B), indicating that the hypoxic procedure used stimulated a hypoxic response in the hippocampus.

To investigate the effect of hypoxia on the Wnt/β-catenin signaling pathway, we evaluated the stabilization of β-catenin and observed a significant increase in its levels with all hypoxic treatments compared to control animals (Figure 1C), suggesting that the canonical Wnt pathway was activated. Importantly, hypoxia induced a mobility shift of Dvl3 (Figure 1A, arrow heads), suggesting that the phosphorylation of Dvl3 was induced, which is normally triggered by the activation of the Wnt pathway due to the binding of a Wnt ligand to Frizzled receptors and to co-receptors (Gao and Chen, 2010). The highest effect on β-catenin levels and Dvl3 phosphorylation was observed after a 24 h exposure to hypoxia, however a clear effect was already observed after 6 h. An increase in c-myc and cyclin D1 levels (Figure 1D), two well-known Wnt target genes (Mann et al., 1999; Hodar et al., 2010), was also observed in response to hypoxia treatment. Altogether, these results suggest that chronic hypoxia induces the activation of the Wnt/β-catenin signaling cascade in the adult hippocampus.

### Chronic hypoxia increases neurogenesis in the subgranular zone (SGZ) of adult mice

**Figure 2:**
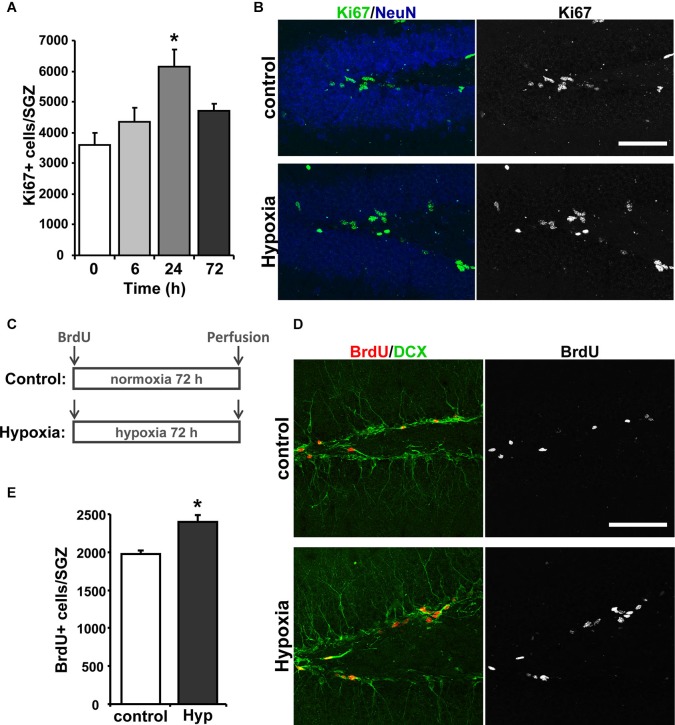
**Hypoxia induces neurogenesis in the hippocampus of adult mice. (A)** Quantification of total number of Ki67 positive (Ki67+) cells in the SGZ of control mice and mice exposed to 6, 24 and 72 h of hypoxia. **(B)** Representative immunofluorescence staining of Ki67 in the hippocampus of control mice and mice exposed to hypoxia for 24 h. Scale bar: 50 µm. **(C)** Schematic representation of the treatment protocol. Control and hypoxia mice received an i.p. injection of 100 mg kg^−1^ BrdU and were then exposed for 72 h to normoxia or hypoxia, respectively, after which were then immediately transcardially perfused. **(D)** Representative double labeling of BrdU and DCX in the hippocampus of control mice and mice exposed to hypoxia for 72 h. Scale bar: 50 µm. **(E)** Quantification of total number of BrdU+ cells in the SGZ of control mice and mice exposed to hypoxia for 72 h. Bars represent mean ± S.E (*n* ≥ 3 mice). * *p* < 0.05.

Next, we evaluated the effect of hypoxia on neurogenesis *in vivo*. First, proliferation was evaluated by immunostaining for the mitotic marker Ki67 (Kee et al., 2002) in the hippocampus of 2-month-old mice exposed to hypoxia for 6, 24 and 72 h. The strongest effect was seen after 24 h of treatment (Figure 2A), which induced a significant increase in total number of Ki67^+^ cells in the SGZ compared to the normoxic control (Figure 2B). These results indicate that chronic hypoxia increases proliferation of hippocampal neural progenitor cells *in vivo*.

To evaluate the differentiation of newborn cells into neurons, mice received a single i.p. injection of 100 mg kg^−1^ BrdU and were exposed to hypoxia or maintained at normoxic conditions for 72 h (Figure 2C), and immunoreactivity for BrdU and the immature neuronal marker doublecortin (DCX) in the hippocampus was investigated (Figure 2D). An increase in the total number of BrdU positive (BrdU^+^) cells was observed in the SGZ of mice exposed to hypoxia (Figure 2E), indicating an increase in cell proliferation. The percentage of the BrdU^+^ cells that were also positive for DCX^+^ was not significantly changed (% BrdU^+^/DCX^+^: control: 69.42 ± 5.20; hypoxia: 70.68 ± 2.91), indicating that the differentiation of BrdU^+^ cells into DCX^+^-neuroblasts was not affected. However, since there is a significant increase of BrdU^+^ cells that differentiate into DCX^+^ cells in mice exposed to hypoxia, these results indicate that chronic hypoxia induces neurogenesis in the hippocampus of adult mice.

### Hypoxia induces neurogenesis in the subgranular zone (SGZ) of double transgenic APPswe/PS1ΔE9 mice

**Figure 3:**
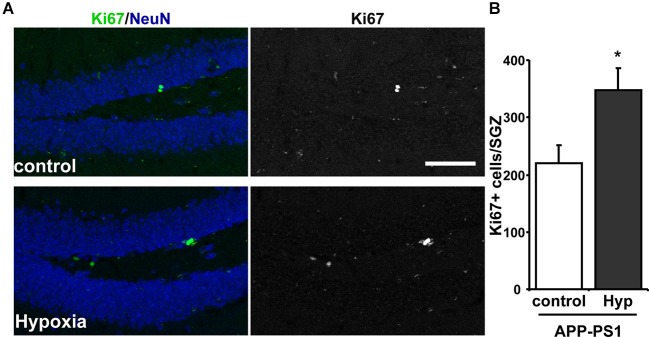
**Increased proliferation in the SGZ of APPswe-PS1ΔE9 mice exposed to hypoxia. (A)** Representative immunofluorescence staining of Ki67 in the hippocampus of control APPswe-PS1ΔE9 mice and APPswe-PS1ΔE9 mice exposed to hypoxia for 72 h. Scale bar: 50 µm. **(B)** Quantification of total number of Ki67 positive (Ki67+) cells in the SGZ of APPswe-PS1ΔE9 mice maintained in control conditions or exposed to hypoxia for 72 h. Bars represent mean ± S.E (*n* = 3 mice). * *p* < 0.05.

Considering the effects on neurogenesis observed in the SGZ of adult wild-type mice, the effect of hypoxia was evaluated in the double transgenic APPswe/PS1ΔE9 mouse model of AD, which shows reduced levels of neurogenesis (Hu et al., 2010; Abbott et al., 2013). For this experiment, 9-month-old APPswe/PS1ΔE9 mice were exposed to 72 h hypoxia, and the effect on cell proliferation was studied by Ki67 immunoreactivity (Figure 3A). A strong increase in total number of Ki67^+^ cells was observed in APPswe/PS1ΔE9 exposed to hypoxia compared to age-matched control APPswe/PS1ΔE9 mice that were not exposed to low oxygen conditions (Figure 3B).

**Figure 4:**
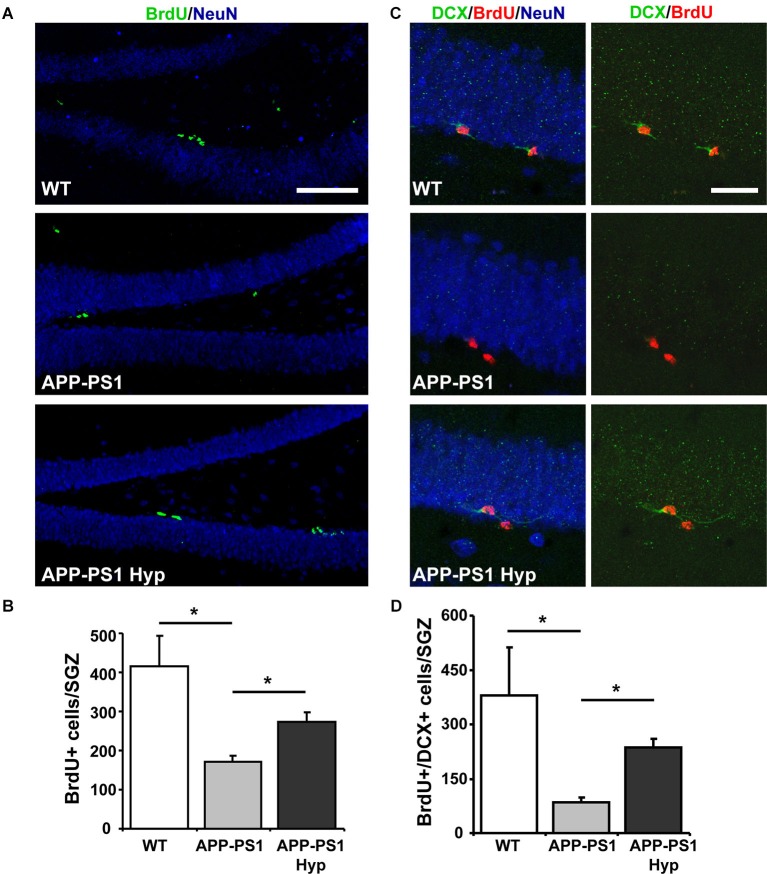
**Hypoxia induces neurogenesis in the hippocampus of APPswe-PS1ΔE9 mice. (A)** Representative double labeling of BrdU and the mature neuronal marker NeuN in the hippocampus of 9-month-old wild-type mice, APPswe-PS1ΔE9 and APPswe-PS1ΔE9 exposed to hypoxia for 72 h. Scale bar: 50 µm. **(B)** Total number of BrdU^+^ cells in the SGZ of all experimental groups. **(C)** Representative double labeling of BrdU and DCX in the same animals. Scale bar: 20 µm. **(D)** Total number of BrdU^+^ cells also positive for DCX (BrdU^+^/DCX^+^) in the hippocampus of all experimental groups. Bars represent mean ± S.E (*n* ≥ 3 mice). * *p* < 0.05.

To assess neurogenesis, mice received a single i.p. injection of 100 mg kg^−1^ BrdU before exposure to hypoxia for 72 h. As a control, age-matched wild-type and APPswe/PS1ΔE9 mice received the BrdU injection but were not exposed to hypoxia and were sacrificed 72 h after BrdU administration. As expected, the total number of BrdU^+^ cells was lower in 9-month-old wild-type (Figures 4A, B) than in 2-month-old wild-type mice (Figure 2D), because of the age-dependent decline in hippocampal neurogenesis (Kuhn et al., 1996; Gould et al., 1999; Leuner et al., 2007; Snyder and Cameron, 2012). In addition, as previously reported (Abbott et al., 2013), a decreased number of BrdU^+^ cells was observed in APPswe/PS1ΔE9 compared to wild-type mice, which was significantly increased after hypoxia (Figures 4A, B). The differentiation of newborn cells into DCX+ neuroblasts and immature neurons, evaluated by double labeling of BrdU and DCX (Figure 4C), was decreased in APPswe/PS1ΔE9 mice compared to age-matched wild-type mice (% BrdU^+^/DCX^+^: wild-type: 68.64 ± 2.656; APPswe/PS1ΔE9: 41.05 ± 6.094), and it was strongly increased in transgenic mice exposed to hypoxia (72.46 ± 4.493). In fact, when analyzing the total number of BrdU^+^/DCX^+^ cells in the hippocampus (Figure 4D), we observed a significant increase in APPswe/PS1ΔE9 mice exposed to hypoxia compared to control APPswe/PS1ΔE9 mice maintained in normoxic conditions (Figure 4D). Altogether, these results indicate that hypoxia stimulates neurogenesis in APPswe/PS1ΔE9 mice.

## Discussion

In the present study, we have determined that *in vivo* exposure to mild hypoxia increases the activation of the Wnt/β-catenin signaling pathway in the hippocampus of adult mice and stimulates cell proliferation and neurogenesis in the SGZ of adult wild-type mice and in the double transgenic APPswe/PS1ΔE9 mouse model of AD.

Hypoxia normally occurs during embryonic stages and it is fundamental for proper neurogenesis during development (Zhu et al., 2005a; Zhang et al., 2011). Moreover, in the adult hippocampus, there are normally low oxygen regions in the SGZ (Mazumdar et al., 2010), indicating a hypoxic microenvironment in the neurogenic niche. Low oxygen stimulates the proliferation and differentiation of embryonic NSC *in vitro* (Studer et al., 2000; Zhao et al., 2008b), and intermittent hypobaric hypoxia increases cell proliferation and neurogenesis in the SVZ and SGZ of adult rats (Zhu et al., 2005b, 2010). HIF-1α has been shown to be critical for the hypoxia-induced proliferation of NSCs *in vitro* and *in vivo* (Zhao et al., 2008b; Mazumdar et al., 2010). The HIF-1α-mediated effect on NSCs involves β-catenin-dependent transcription since HIF-1α increases the expression of Lef1 and TCF1 (Mazumdar et al., 2010), the nuclear partners of β-catenin for the activation of Wnt target genes (Logan and Nusse, 2004). Here we determined that concomitantly with the increase in the levels of HIF-1α, exposure to 10% O_2_ stimulated Dvl3 phosphorylation, β-catenin stabilization and the transcription of Wnt target genes in the hippocampus of adult mice. These results indicate that hypoxia not only regulates transcriptional activation of β-catenin, but also induces the activation of the Wnt/β-catenin signaling cascade *in vivo*, which has not been previously reported. The mechanism involved may comprise the increased transcription of Wnt ligands and/or Frizzled receptors. In mammals, 19 Wnt ligands and 10 Frizzled receptors have been identified, many of them being present in the adult brain (Shimogori et al., 2004; Chen et al., 2006; Chacon et al., 2008). Also, hypoxia may regulate the levels of secreted inhibitors of the Wnt signaling pathway such as Dickkopf 1 (Dkk1) and soluble Frizzled-related protein 3 (sFRP3), both recently described as negative regulators of adult hippocampal neurogenesis that can be regulated under certain physiological conditions (Jang et al., 2013; Seib et al., 2013). Whether or not exposure to hypoxic conditions regulates the expression of Wnt signaling components will have to be explored further.

We also determined that exposure to chronic hypoxia induced cell proliferation in the SGZ of adult mice as determined by BrdU incorporation and Ki67 staining. Importantly, the neuronal differentiation of newborn cells was not changed, indicating that hypoxia-induced proliferation results in increased newborn neurons. The hypoxia-induced proliferation was also observed in the SGZ of a double transgenic mouse model of AD. AD is a neurodegenerative disease characterized by progressive deterioration of cognitive abilities. Two neuropathological hallmarks of AD are the extracellular senile plaques mainly composed of amyloid-β (Aβ) peptide and intracellular neurofibrillary tangles formed by hyperphosphorylated tau protein (Castellani et al., 2010; Ballard et al., 2011; Mandelkow and Mandelkow, 2012). The double transgenic APPswe-PS1ΔE9 mice at the age used in the present study show most histopathological markers of AD (Inestrosa et al., 2011), and show decreased levels of neurogenesis as previously reported (Abbott et al., 2013) and as observed here. Hypoxia strongly stimulated proliferation and neuronal differentiation in AD mice, indicating that hypoxia could stimulate this process in the diseased brain.

The possibility to stimulate neurogenesis in the adult brain may offer an exciting alternative for brain repair. Considering the described roles of neurogenesis in learning and memory (Deng et al., 2010; Koehl and Abrous, 2011; Marin-Burgin and Schinder, 2012), the hypoxia-induced activation of progenitor cells in the adult hippocampus may help to ameliorate the cognitive decline associated to neurodegenerative diseases. Not only is the effect of hypoxia on neurogenesis of therapeutic interest. Our findings indicating that mild hypoxia induces the activation of the Wnt signaling pathway in the adult brain may also have therapeutic benefits. The dysfunction of the Wnt/β-catenin signaling pathway has been linked to neurodegenerative disorders such as schizophrenia, autism and AD (Moon et al., 2004; Lovestone et al., 2007; Inestrosa et al., 2012). Several studies have shown that Wnt signaling components are altered in AD (De Ferrari and Inestrosa, 2000; Caricasole et al., 2004; Ghanevati and Miller, 2005; De Ferrari et al., 2007; Toledo and Inestrosa, 2010), and that Wnt signaling activation has neuroprotective properties against the toxicity of Aβ peptide (De Ferrari et al., 2003; Alvarez et al., 2004; Chacon et al., 2008). Therefore, the hypoxia-induced activation of the Wnt/β-catenin signaling pathway may be relevant for the treatment of AD and other pathologies associated with impaired Wnt signaling.

## Conflict of interest statement

The authors declare that the research was conducted in the absence of any commercial or financial relationships that could be construed as a potential conflict of interest.
